# What Is Man’s Natural Food?

**Published:** 1896-10

**Authors:** Charles E. Page

**Affiliations:** Boston


### SELECTIONS.

### What is Man's Natural Food?

BY CHARLES E. PAGE, M.D., BOSTON.

A few years ago the writer contributed an article on the topic of natural diet to one of our leading medical journals, taking the ground that nuts and fruits were not only the original diet of human beings, but that they are to-day, by all means, the most wholesome and nutritious of all foods. Nuts of all kinds, the fresh, juicy fruits, as oranges, peaches, apples, etc., the date, fig, grape (raisins), etc.—all these are relished by mankind, the world over, and supply every element required for perfect nourishment, and, generally speaking, all these foods that are absolutely essential to life and health are obtainable by the greater proportion of the people. Impecunious persons can not indulge in strawberries at a dollar a box, peaches at five dollars a basket, or other fancy varieties at fancy prices, to be sure, but, assuredly, all persons who can pay from fifteen to twenty cents a pound for beef, as we know is true of no end of wretchedly-poor people in the mistaken notion that a large share of animal food is essential to health, or, perhaps, in the idea that they must imitate the diet of the rich—can pay as much per pound for English walnuts, or other varieties of nut-food; and many would be inclined to do this if they were fortunate enough to know that nut-food is far more nutritious and a better balanced diet than flesh-food at any price.

That wonderfully-muscular annimal, the squirrel, that carries himself with such rapidity, almost like lightning, to the top of the tallest tree—does he not build his muscle from nuts? In winter this is his chief food, and in spring he lives on the fresh buds and green things, comparable to our fresh fruits, early vegetables, lettuce, etc. Almost every one is fond of nuts of all kinds, and we know that nuts and raisins are served as the last course of swell dinners, probably because they are so delicious that they tempt the appetite, even after the diners are fed to repletion. And this fully accounts for their alleged indigestibility; they are usually eaten after an excess of food has already been taken, or they are taken in excess because so delicious that "one doesn't know when to stop." Under like conditions breast-milk, unquestionably, the natural diet of the nursling, proves "indigestible." Too much of a good thing is bad.

In acknowledging the receipt of a reprint of the article mentioned above Prof. Edward Atkinson, the eminent student of household economics, writes: "You may be interested to learn that Sir Isaac Holden, probably, or almost certainly, the inventor of the lucifer match and absolutely the inventor of the largest and most successful wool-combing machine, now very rich and nearly the Nestor of Parliament (about 86), believes that nuts and fruits are the natural food, and by limiting his diet mainly thereto he may expect to extend the duration of life to one hundred and twenty-five years. His place, in Yorkshire, is a very interesting one, especially his acres of glazed fruit-gardens on the ridges of Yorkshire."

The natural diet question is, at the present time, attracting a great deal of attention, and throughout Europe, England, and even here in America, the propaganda of "the natural diet" has, within the past few years, acquired quite an impetus through the intelligent efforts of the Drs. Densmore, of London and New York. Rich enough to enable them to do so, they have established a health journal, *Natural Food*, publishing it monthly at a cheap rate, in London. The evidence of the benefits arising from this natural diet, furnished in the columns of the modest little journal referred to, would be quite surprising to any one not aleady a convert to the theory that the nuts and fruits are the best foods for nourishing the body in health, and best calculated to aid in the cure of many forms of chronic disease.

The nut is so "hearty," contains such an amount of solid material, that a novice might easily take an excess of nutriment, unless admonished as to amount. I once delighted the heart of a lady patient by prescribing nuts as a part of her diet, cautioning her against taking an excess. Doubtless forgetting my advice as to quantity she bought a pound of English walnuts and ate most of them at one meal. Naturally enough she reported the next week that she was "sick of walnuts," and couldn't eat them any more. A few ounces of nut-meat, with a large proportion of one of the sweet fruits, as dates or raisins, make a full dinner. A hearty lunch may be made from, say, two or three dozen dates with a sip of milk with each, making a "sweet pudding" that no *chef* on earth can equal for wholesomeness or excel for toothsomeness.

